# Coupling Bacterial Community Assembly to Microbial Metabolism across Soil Profiles

**DOI:** 10.1128/mSystems.00298-20

**Published:** 2020-06-09

**Authors:** Lu Luan, Chao Liang, Lijun Chen, Haotian Wang, Qinsong Xu, Yuji Jiang, Bo Sun

**Affiliations:** a State Key Laboratory of Soil and Sustainable Agriculture, Institute of Soil Science, Chinese Academy of Sciences, Nanjing, China; b Institute of Applied Ecology, Chinese Academy of Sciences, Shenyang, China; c College of Life Science, Nanjing Normal University, Nanjing, China; d University of Chinese Academy of Sciences, Beijing, China; Lawrence Berkeley National Laboratory

**Keywords:** bacterial community assembly, microbial metabolism, bacterial interactions, migration rate, environmental filtering, soil profile

## Abstract

Soil microbial community assembly is crucial for understanding the mechanisms of microbial communities that regulate ecosystem-level functioning. The relative contributions of stochastic and deterministic processes to microbial community assembly remain poorly defined, and major questions exist concerning the soil organic carbon (SOC) dynamics of microbial community assembly in deep soil. Here, the bacterial community assembly processes were explored across five soil profile depths (up to 80 cm) during a 15-year field experiment involving four fertilization regimes. We found that the bacterial community assembly was initially governed by deterministic selection in topsoil but was progressively structured by increasing stochastic dispersal with depth. The migration rate (*m*) and β-null deviation pattern supported the hypothesis of a relatively greater influence of dispersal in deep soil, which was correlated with bacterial community assembly by stochastic processes. These changes in the entire community assembly reflected consistent assembly processes of the two most dominant phyla, *Acidobacteria* and *Chloroflexi*. Structural equation modeling showed that soil features (pH and total phosphorus) and bacterial interactions (competition and network complexity) were significantly related to bacterial community assembly in the 0-to-10-cm and 10-to-20-cm layers. Partial Mantel tests, structural equation modeling, and random forest modeling consistently indicated a strong and significant correlation between bacterial community assemblages and SOC dynamics, implying that bacterial assembly processes would potentially suppress SOC metabolism and mineralization when the contributions of stochastic dispersal to communities increased in deeper layers. Our results have important implications for integrating bacterial community assembly processes into the predictions of SOC dynamics.

**IMPORTANCE** We have provided a framework to better understand the mechanisms governing the balance between stochastic and deterministic processes and to integrate the shifts in community assembly processes with microbial carbon metabolism. Our study reinforced that environmental filtering and bacterial cooccurrence patterns influence the stochastic/deterministic continuum of soil bacterial community assembly and that stochasticity may act through deeper soil layers to influence carbon metabolism. Delineating theoretically the potential linkages between community assembly and SOC dynamics across a broad range of microbial systems represents an interesting topic for future research.

## Abstract (precis)

We have provided a framework to better understand the mechanisms governing the balance between stochastic and deterministic processes and to integrate the shifts in community assembly processes with microbial carbon metabolism. Our study reinforced that environmental filtering and bacterial cooccurrence patterns influence the stochastic/deterministic continuum of soil bacterial community assembly and that stochasticity may act through deeper soil layers to influence carbon metabolism. Delineating theoretically the potential linkages between community assembly and SOC dynamics across a broad range of microbial systems represents an interesting topic for future research.

## INTRODUCTION

Understanding the mechanisms controlling microbial community assembly is central but poorly studied in microbial ecology. Generally, fundamental ecological processes can be grouped into two mutually nonexclusive determinants of microbial community assembly, i.e., deterministic and stochastic processes, which simultaneously play important roles in the maintenance of species composition from local to global scales (1–4). Determinism is largely dictated by selection, including environmental filtering and various biological interactions (e.g., competition, mutualism, and predation), and thereby determines the fitness and abundance of species (1, 2). Alternatively, stochasticity is associated with the random ecological drift, probabilistic dispersal, and evolutionary diversification that generate divergence in the patterns of community composition (3, 4). Studies have now progressed toward quantitatively estimating the relative influences of ecological processes in bacterial community assembly across natural ecosystems (5–7). As a consequence, there are many associated models, such as the beta-null model (8), neutral model (9), and phylogenetic sampling theory (10). However, none of the models mentioned above can simultaneously and quantitatively separate the contributions of each ecological process to community assemblages. Recently, Stegen’s null modeling approach was proposed to estimate the relative influences of ecological components, such as selection, dispersal, and drift, within Vellend’s framework at the scale of a metacommunity (2, 11). In the null model, community assembly is divided into five main categories, namely, variable selection, homogeneous selection, homogeneous dispersal, dispersal limitation, and undominated processes (“undominated” refers to compositional differences between communities that are due to a mixture of stochastic organismal movements and stochastic birth-death events [11]). This emerging model has therefore been widely applied to understand microbial community assembly in various ecosystems (5–7).

It has been universally acknowledged that bacterial community assembly is greatly affected by the combination of abiotic environmental filtering and biotic interactions. Environmental variations are prerequisites of species niche differentiation, which enables distinct bacteria to obtain sufficient resources and survive under diverse environmental conditions (12). Numerous works have demonstrated that the assembly processes of microbial communities are driven by a wide range of edaphic parameters, such as soil organic matter (5), soil pH (7), and total phosphorus (13). Biotic interactions (competition and corporation) are other determinants of microbial community assembly (3), and cooccurrence network analysis is a powerful method to elucidate these interactions (14, 15). In fact, in-depth investigations of cooccurrence networks are increasingly intriguing for microbial ecologists because the cooccurrence patterns in the highly complex bacterial communities may serve as indicators of niche differentiation and overlap for inferring potential biological interactions and linking ecological processes to community assembly (14, 16). Soil profiles with environmental transition present a unique opportunity for examining community assembly processes, as these profiles experience variations in physicochemical properties and bacterial community compositions across tractable spatial scales (17, 18). As levels of nutrients and oxygen generally fluctuate throughout a soil profile, the corresponding changes are reflected in the diversity and cooccurrence networks of bacterial communities (18). Thus, an issue exists as to whether depth gradients influence the relative levels of importance of stochastic and deterministic processes. The comprehensive perspective of assembly rules will deepen the understanding of the main factors underpinning observed community assembly throughout soil layers.

The deep soil organic carbon (SOC) reservoir (>30 cm in depth) is the primary component of the SOC pool within the top meter, where more than half of the SOC stock is located in subsoil horizons (19). Although agricultural management practices strongly control SOC dynamics throughout the soil profile (20), how to determine the biological processes involved in the changes in deep soil carbon remains a challenge. Traditional studies have focused on the important roles of microbial biomass and community composition in functioning at deep depths (21, 22), while recently emerging theories have highlighted the novel mechanisms of microbial community assembly processes in mediating microbial carbon metabolism (23). Microbial community assembly processes impose constraints on community membership and subsequently determine microbial functioning. The direction and extent of community assembly processes governing microbial carbon metabolism depend heavily on myriad spatial scales (24, 25). The variations in the microbial assembly processes due to selective pressures and dispersal scenarios couple with rapid shifts in the rates of microbial carbon metabolism (23, 25). However, our comprehension of how bacterial assembly processes influence carbon metabolism is still nascent. To a large extent, the lack of knowledge about microbial community assembly in terms of influencing carbon metabolism limits the understanding of SOC dynamics across the soil profile.

Here, we aimed to investigate the impact of community assembly processes on carbon metabolism and SOC mineralization across soil profiles (0 to 80 cm). Specifically, the main scientific questions addressed in this study were as follows. (i) How do the relative influences of assembly processes regulate soil bacterial community changes throughout the soil profile? (ii) How do soil properties and bacterial cooccurrence cooperatively affect bacterial community assembly processes? (iii) What are the biological mechanisms of community assembly patterns mediating carbon metabolism and SOC mineralization? To address these questions, we conducted a 15-year field experiment that involved four fertilization regimes along five soil profile depths in a red soil (Acrisol). High-throughput sequencing technology coupled with network analysis was used to evaluate the assembly processes and cooccurrence networks of the bacterial communities throughout the studied soil layers. The various carbon substrate utilization profiles were determined via the use of Biolog EcoPlates as a measure of microbial carbon metabolism, and SOC mineralization was measured by cumulative CO_2_ emissions in the microcosms. Through substantial analyses, our results indicated a strong and significant correlation between bacterial community assemblages and SOC dynamics, implying that the bacterial assembly processes would potentially suppress SOC metabolism and mineralization when the contributions of stochastic dispersal to communities increased in the deeper layers.

## RESULTS

### Soil physiochemical properties and microbial carbon metabolic profiles.

**FIG 1:**
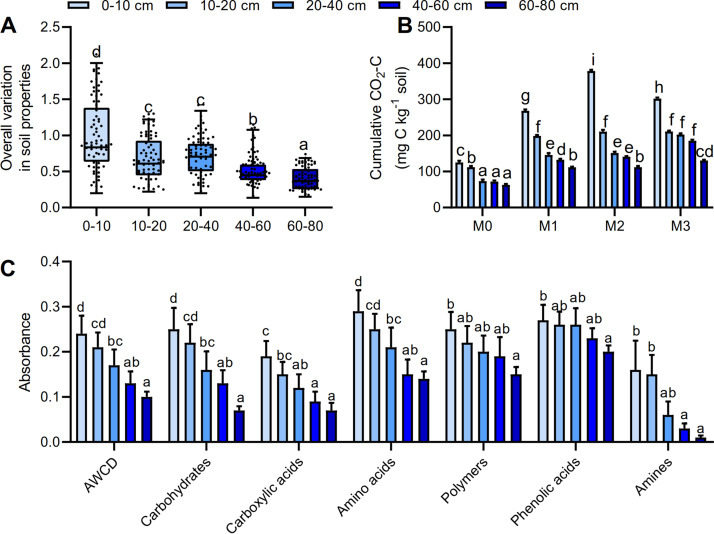
Environmental variation and the soil carbon dynamics across soil profile. (A) Box plot showing the overall variation in soil properties according to variance-covariance matrices based on all soil properties in each soil layer. (B) SOC mineralization is indicated by cumulative CO_2_−C. M0, no manure; M1, low manure; M2, high manure; M3, high manure plus lime. (C) Carbon metabolic activities are reflected by the average well color development (AWCD). The carbon sources are further subdivided into six group substrates, including carbohydrates, carboxylic acids, amino acids, polymers, phenolic acids, and amines. Bars with different lowercase letters indicate significant differences (*P < *0.05) across soil profiles, as revealed by one-way ANOVA with Turkey’s *post hoc* test. Each average value was calculated from 12 samples of each soil layer.

SOC, pH, total nitrogen (TN), and total phosphorus (TP) declined sharply across the soil layers (see Fig. S1 in the supplemental material). The soil moisture content (SMC), total potassium (TK), and ammonia nitrogen (NH_4_−N) exhibited a unimodal pattern throughout the soil layers, peaking in the 40-to-60-cm layer. In contrast, nitrate nitrogen (NO_3_−N) was significantly higher in the 0-to-10-cm and 20-to-40-cm layers than in the 10-to-20-cm and 60-to-80-cm layers. Two-way permutational multivariate analysis of variance (PERMANOVA) revealed that soil depth had a stronger effect on the soil characteristics than the fertilization treatments (see Table S1 in the supplemental material; *P < *0.001). Coefficients of variation for soil pH, TP, SOC, and TK were higher in the 0-to-10-cm layer than in the other layers and tended to decrease with soil depth (Fig. S1). The environmental variation (variance-covariance matrix) of the soil properties in the 0-to-10-cm layer was significantly higher than in the subsoil (10 to 80 cm; Fig. 1A) (*P < *0.05). The microbial metabolic activities reflected by the average well color development (AWCD) and SOC mineralization were significantly distinguished by soil depth (Fig. 1B and C) (*P < *0.05) such that, compared with the four other layers, the 0-to-10-cm layer was characterized by the largest values of AWCD and SOC mineralization. The utilization of carbohydrates, carboxylic acids, amino acids, and amines followed the same trend as that of the AWCD for the whole plate (*P < *0.05). However, no significant differences were observed in the utilization of two different guilds, polymers (*P = *0.350) and phenolic acids (*P = *0.329).

10.1128/mSystems.00298-20.1 FIG S1 The physicochemical properties in soil depth profiles, including pH (A), soil organic carbon (SOC) (B), total phosphorus (TP) (C), total potassium (TK) (D), soil moisture content (SMC) (E), total nitrogen (TN) (F), ammonia nitrogen (NH_4_–N) (G), and nitrate nitrogen (NO_3_–N) (H). Bars with different lowercase letters indicate significant differences (*P < *0.05) across soil profiles, as revealed by one-way ANOVA with Turkey’s *post hoc* test. The number above the horizontal line indicates the coefficient of variation of the environmental factor in each soil layer. M0, no manure; M1, low manure; M2, high manure; M3, high manure plus lime. Download FIG S1, PDF file, 0.02 MB. Copyright © 2020 Luan et al. 2020 Luan et al. This content is distributed under the terms of the Creative Commons Attribution 4.0 International license.

10.1128/mSystems.00298-20.7 TABLE S1 Two-way PERMANOVA showing the effects of soil depth and fertilization treatments on soil properties, the bacterial community, carbon metabolism, and SOC mineralization. Download Table S1, PDF file, 0.2 MB. Copyright © 2020 Luan et al. 2020 Luan et al. This content is distributed under the terms of the Creative Commons Attribution 4.0 International license.

### Biomass, diversity, and composition of the bacterial communities.

The 0-to-40-cm layer presented substantially higher bacterial biomass and diversity than the 40-to-60-cm and 60-to-80-cm layers (Fig. S2A to C) (*P < *0.05). At the phylum/class level, the bacterial communities were predominated by *Chloroflexi* (26.7%), *Acidobacteria* (18.1%), *Actinobacteria* (16.4%), and *Alphaproteobacteria* (9.3%), followed by *Firmicutes* (5.3%), Deltaproteobacteria (4.3%), *Betaproteobacteria* (3.8%), and *Gammaproteobacteria* (3.1%), across all samples (Fig. S2D). Principal-coordinate analysis (PCoA) revealed that the bacterial communities were clearly segregated by soil depth (Fig. S2E) (*P < *0.001). In terms of relative abundance, *Acidobacteria* abundance was significantly lower in the topsoil than in the subsoils, whereas the abundances of *Alphaproteobacteria* and Deltaproteobacteria were significantly higher in the topsoil than in the subsoils (Fig. S3) (*P < *0.05). Furthermore, *Chloroflexi* abundance was significantly higher in the 10-to-20-cm layer than in the 0-to-10-cm layer, while *Actinobacteria* and *Firmicutes* exhibited the opposite trend. Two-way PERMANOVA indicated that soil depth yielded a substantial impact on the bacterial biomass and diversity, while fertilization treatments had less of an influence (Table S1, *P < *0.001).

10.1128/mSystems.00298-20.2 FIG S2 Soil bacterial biomass (A) was determined by 16S rRNA gene copy number, and soil bacterial diversity was determined by Shannon index (B) and Chao1 richness (C) across soil depth profiles. Calculation of values on the basis of Shannon index and Chao1 richness is based on OTU tables rarified to the same sequencing depth. Bars with different lowercase letters indicate significant differences (*P < *0.05) as revealed by one-way ANOVA with Turkey’s *post hoc* test. (D) Taxonomic composition of soil bacterial communities across soil depth profiles. The values representing the relative abundances of bacterial communities were determined on the basis of the proportion of 16S rRNA gene sequences. (E) Soil depth profiles well separate the bacterial community compositions based on Bray-Curtis dissimilarity using principal-coordinate analysis. The percentage values indicated in parentheses denote the proportion of variation explained by each axis. M0, no manure; M1, low manure; M2, high manure; M3, high manure plus lime. Download FIG S2, PDF file, 0.3 MB. Copyright © 2020 Luan et al. 2020 Luan et al. This content is distributed under the terms of the Creative Commons Attribution 4.0 International license.

10.1128/mSystems.00298-20.3 FIG S3 The distribution of dominant phyla across soil profiles. (A) Bars with different lowercase letters indicate significant differences (*P* < 0.05) within the bacterial phyla, as revealed by one-way ANOVA with Turkey’s *post hoc* test. (B) Relative abundances of eight dominant phyla across soil profiles and fertilization treatments. M0, no manure; M1, low manure; M2, high manure; M3, high manure plus lime. Download FIG S3, PDF file, 0.02 MB. Copyright © 2020 Luan et al. 2020 Luan et al. This content is distributed under the terms of the Creative Commons Attribution 4.0 International license.

### Assembly processes of the bacterial communities.

**FIG 2:**
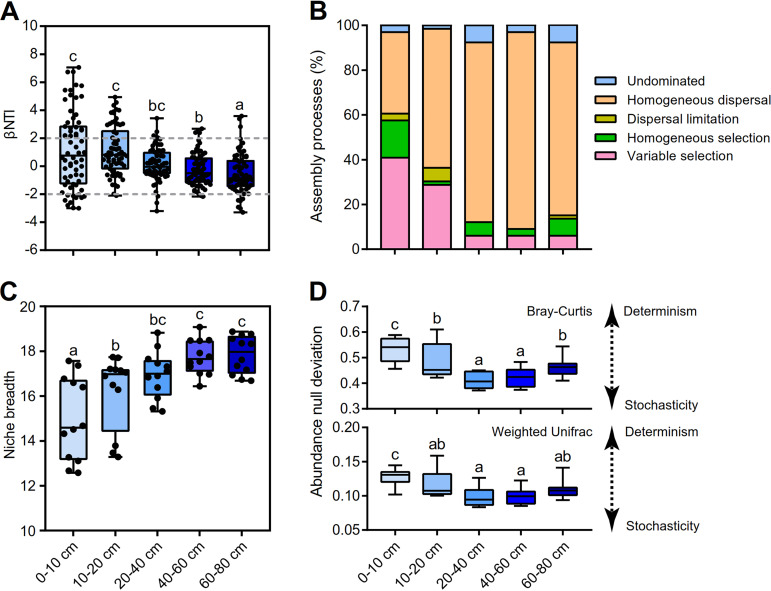
The bacterial community assembly processes across soil profiles. (A) The values of the weighted beta nearest taxon index (βNTI) for soil bacterial communities present. Horizontal dashed gray lines indicate upper and lower significance thresholds at βNTI = +2 and −2, respectively. (B) The percentage of turnover in soil bacterial community assembly, governed principally by deterministic processes (homogeneous and variable selection), stochastic processes (dispersal limitation and homogenizing dispersal), or undominated processes. (C) Habitat niche breadth of the bacterial communities. (D) The deviations of Bray-Curtis dissimilarities and weighted UniFrac distances were calculated to separate the community assembly into deterministic and stochastic processes. A β-diversity deviation value closer to zero indicates higher stochasticity, whereas a β-diversity deviation value closer to 1 or −1 indicates higher deterministicity. Bars with different lowercase letters indicate significant differences within the bacterial phyla (*P < *0.05) across soil profiles, as revealed by one-way ANOVA with Turkey’s *post hoc* test. Values for each soil layer were calculated from 12 samples.

The metric of the weighted bacterial community assembly (βNTI) provided insights into the potential roles of both deterministic and stochastic forces in the phylogenetic community dynamics of bacterial communities. Two-way PERMANOVA showed that bacterial community assembly (βNTI) was more pronouncedly influenced by soil depth (*F* = 106.59, *R*^2^ = 0.59, *P < *0.001) than by fertilization treatments (*F* = 9.25, *R*^2^ = 0.11, *P < *0.001) (Table S1). We observed that the βNTI distributions differed significantly across the soil depth [*F*_(4, 325)_ = 5.22, *P < *0.001], from deterministic community assembly (|βNTI| > 2) to stochastic assembly (|βNTI| < 2) (Fig. 2A). Specifically, the contribution of deterministic processes to community assembly sharply decreased across the soil profile and peaked in the 0-to-10-cm layer. The deterministic processes of variable selection contributed 40.9% to the community assembly, followed in importance by homogeneous selection (16.7%) (Fig. 2B). However, the stochastic processes remained dominant in shaping the bacterial community assembly in the 10-to-80-cm layers. The stochastic process of homogeneous dispersal was responsible primarily for the assembly and turnover of the soil bacterial communities (43% to 91%). We further observed the relative influences of the stochastic and deterministic ecological processes that mediated the assembly of the dominant phyla (Fig. S4). Consistent with the entire bacterial community assembly, the βNTI values for both *Acidobacteria* and *Chloroflexi* revealed that the relative influence of stochastic processes gradually increased with increasing soil depth. In contrast, the contribution of stochastic assembly for *Actinobacteria*, *Alphaproteobacteria*, *Betaproteobacteria*, Deltaproteobacteria, *Firmicutes*, and *Gammaproteobacteria* was dominant throughout the soil profile.

10.1128/mSystems.00298-20.4 FIG S4 The percentage of turnover in the assembly processes of dominant bacterial phyla, including *Acidobacteria* (A), *Actinobacteria* (B), *Alphaproteobacteria* (C), *Betaproteobacteria* (D), *Chloroflexi* (E), Deltaproteobacteria (F), *Firmicutes* (G), and *Gammaproteobacteria* (H), governed primarily by deterministic processes (homogeneous and variable selection), stochastic processes (dispersal limitation and homogenizing dispersal), or undominated processes across soil depth profiles. Download FIG S4, PDF file, 0.2 MB. Copyright © 2020 Luan et al. 2020 Luan et al. This content is distributed under the terms of the Creative Commons Attribution 4.0 International license.

To explore the relative levels of importance of stochastic processes in bacterial community assembly along soil profiles, a neutral model of community assembly combined with abundance-based β-null approaches was further fitted to distinguish deterministic and stochastic processes. The habitat niche breadth values and migration rates (*m*) of soil bacterial communities showed a sharply increasing trend with increased soil depths (Fig. 2C; see also Table S2). However, the β-null deviations of Bray-Curtis dissimilarity and weighted UniFrac distance were statistically significantly higher in the 0-to-10-cm layers than in the 10-to-80-cm layers (Fig. 2D) (*P < *0.05), indicating that the bacterial community assembly was a more deterministic process in the 0-to-10-cm layers than in the 10-to-80-cm layers. At the phylum level, the β-null deviation value, habitat niche breadth, and *m* values for *Acidobacteria* and *Chloroflexi* followed a trend similar to that seen with the entire bacterial community (Fig. S5 and S6; see also Table S2). *Actinobacteria*, *Alphaproteobacteria*, *Betaproteobacteria*, Deltaproteobacteria, *Firmicutes*, and *Gammaproteobacteria* exhibited higher habitat niche breadth and *m* values but lower β-null deviations across soil depths.

10.1128/mSystems.00298-20.5 FIG S5 Deviations from abundance-weighted β-null models (Bray-Curtis dissimilarity and weighted UniFrac distance) were combined to assess the relative changes in deterministic and stochastic processes at the phylum level, including *Acidobacteria* (A), *Actinobacteria* (B), *Alphaproteobacteria* (C), *Betaproteobacteria* (D), *Chloroflexi* (E), Deltaproteobacteria (F), *Firmicutes* (G), and *Gammaproteobacteria* (H). A β-diversity deviation value closer to zero indicates higher stochasticity, whereas a β-diversity deviation value closer to 1 or −1 indicates higher deterministicity. Bars with different lowercase letters indicate significant differences (*P < *0.05) within the bacterial phyla, as revealed by one-way ANOVA with Turkey’s *post hoc* test. Download FIG S5, PDF file, 0.2 MB. Copyright © 2020 Luan et al. 2020 Luan et al. This content is distributed under the terms of the Creative Commons Attribution 4.0 International license.

10.1128/mSystems.00298-20.6 FIG S6 Box plots illustrating habitat niche breadth at the phylum level, including *Acidobacteria* (A), *Actinobacteria* (B), *Alphaproteobacteria* (C), *Betaproteobacteria* (D), *Chloroflexi* (E), Deltaproteobacteria (F), *Firmicutes* (G), and *Gammaproteobacteria* (H). Bars with different lowercase letters indicate significant differences (*P < *0.05) within the bacterial phyla, as revealed by one-way ANOVA with Turkey’s *post hoc* test. Download FIG S6, PDF file, 0.2 MB. Copyright © 2020 Luan et al. 2020 Luan et al. This content is distributed under the terms of the Creative Commons Attribution 4.0 International license.

10.1128/mSystems.00298-20.8 TABLE S2 Parameters and fits of neutral models that consider the influence of drift with the influence of dispersal across five soil profiles. Download Table S2, PDF file, 0.4 MB. Copyright © 2020 Luan et al. 2020 Luan et al. This content is distributed under the terms of the Creative Commons Attribution 4.0 International license.

### Cooccurrence patterns in the bacterial networks.

**FIG 3:**
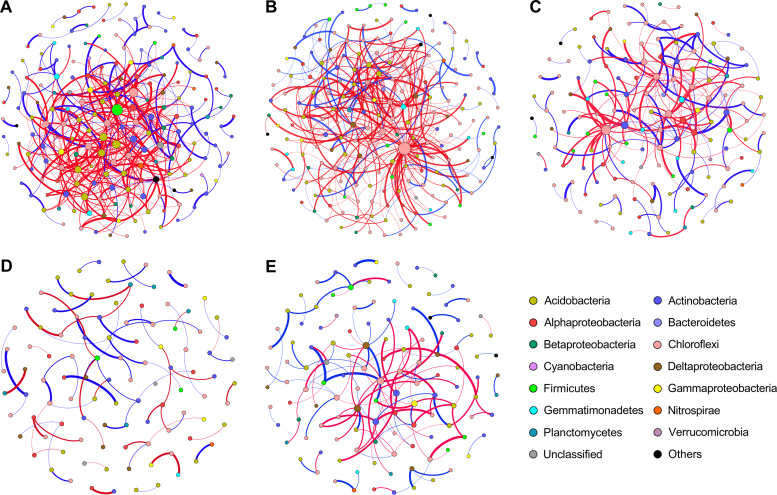
The bacterial cooccurring networks across soil profiles. The networks of cooccurring bacterial OTUs were determined for five soil depth layers, including 0 to 10 cm (A), 10 to 20 cm (B), 20 to 40 cm (C), 40 to 60 cm (D), and 60 to 80 cm (E), based on correlation analysis. For each panel, a connection stands for a strong correlation coefficient (*r*) greater than 0.6 or less than −0.6 and a *P* value of <0.01. The cooccurring networks are colored by phylum/class. The size of each node is proportional to the number of connections (i.e., degree), and the thickness of each connection between two nodes (i.e., edge) is proportional to the value of correlation coefficients. Blue edges indicate positive relationships between two individual nodes, while red edges indicate negative relationships. Each network was constructed from 12 samples.

The cooccurrence patterns were analyzed to explore the potential roles of bacterial interactions in community assembly processes using network analysis. The topological characteristics of the bacterial networks were calculated to decipher the complex cooccurrence patterns among bacteria. The total number of edges and percentage of negative correlations (PNC) tended to decrease as the soil depth increased (Fig. 3; see also Table S3). Structural analysis revealed that the stochastic cooccurrence pattern was prominent in the 20-to-80-cm layers, which was indicated by the inconsistency between the obtained (*O*) and random (*R*) cooccurring incidences in the bacterial communities. Five dominant phyla, *Acidobacteria*, *Actinobacteria*, *Betaproteobacteria*, *Firmicutes*, and *Gammaproteobacteria*, tended to cooccur lower at a lower ratio (*O*/*R* < 1) than expected by random associations, taking the phylum frequency and random expectations into account (Table S4). Similar patterns were also identified for *Chloroflexi* (*O*/*R* = 0.87 to 0.95) in the 40-to-60-cm and 60-to-80-cm layers.

10.1128/mSystems.00298-20.9 TABLE S3 Topological properties of the bacterial cooccurrence networks across soil profiles. Download Table S3, PDF file, 0.3 MB. Copyright © 2020 Luan et al. 2020 Luan et al. This content is distributed under the terms of the Creative Commons Attribution 4.0 International license.

10.1128/mSystems.00298-20.10 TABLE S4 The incidence of cooccurrence for bacterial intraphyla by random association in five soil layers was expected. The observed cooccurring incidence (*O*) of the dominant phyla was estimated by determination of the relative percentages of the number of observed edges between them in the respective networks, while the randomly cooccurring incidence (*R*) was theoretically calculated by considering the phylum/class frequencies [*F*(Node1), *F*(Node2)] and random association. Download Table S4, PDF file, 0.3 MB. Copyright © 2020 Luan et al. 2020 Luan et al. This content is distributed under the terms of the Creative Commons Attribution 4.0 International license.

### Potential important predictors of bacterial community assembly and carbon metabolism.

**FIG 4:**
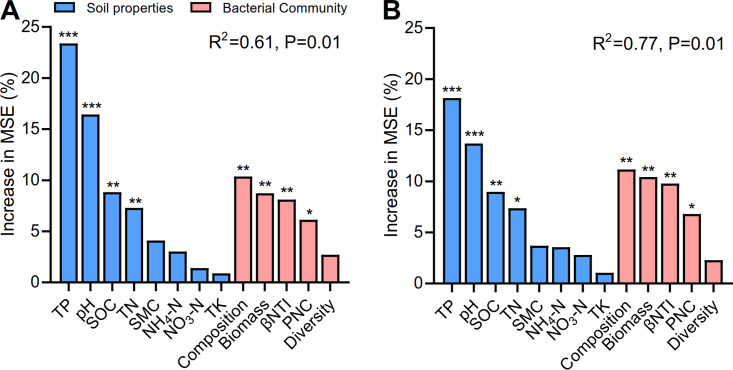
Mean predictor importance (percent increased mean square error, MSE) of carbon metabolism and soil organic carbon (SOC) mineralization. (A) Soil carbon metabolic activities are reflected by the average well color development (AWCD). (B) SOC mineralization is indicated by cumulative CO_2_-C. Soil properties include pH, SOC, total phosphorus (TP), total nitrogen (TN), total potassium (TK), ammonia nitrogen (NH_4_−N), nitrate nitrogen (NO_3_−N), and soil moisture content (SMC). The bacterial community data represent biomass, diversity (Shannon index), composition (Bray-Curtis dissimilarity), assembly processes (βNTI, the weighted beta nearest taxon index), and the cooccurring networks (PNC, the percentage of negative correlations). Significance levels of predictors are indicated as follows: ***, *P < *0.001; **, *P < *0.01; *, *P < *0.05.

The partial Mantel test showed that the βNTI scores were significantly correlated with TP (*R *= 0.53, *P < *0.001) and pH (*R *= 0.50, *P < *0.001), as well as with carbon metabolism (*R *= 0.43, *P < *0.001) and SOC mineralization (*R *= 0.39, *P < *0.01) (Table 1). Random forest modeling was performed to separate and assess the important predictors of the bacterial community assembly and carbon metabolic profiles across soil layers (Fig. 4). The models for carbon metabolic activities and SOC mineralization were significant at the 0.01 level (*R*^2^ = 0.61 and 0.77). We observed that TP and pH were the two most important predictors of carbon metabolism (23.4% and 16.4%) and soil mineralization (18.1% and 13.7%), respectively. Furthermore, the community composition (10.4% and 11.2%), biomass (8.7% and 10.4%), assembly processes (8.1% and 9.8%), and PNC (6.1% and 6.8%) of the bacterial community contributed significantly to carbon metabolism and soil mineralization, respectively.

**TABLE 1:** Correlations among soil properties, bacteria community, and carbon metabolic activities and SOC mineralization^*a*^

Parameter	Correlation value									
	pH	TP	SOC	TN	TK	SMC	NO_3_–N	NH_4_–N	AWCD	SOC mineralization
Biomass	0.44***	0.51***	−0.1	0.44**	0.11	−0.68***	0.22	−0.31*	0.47***	0.64***
Shannon	0.16	0.04	0.16	−0.03	−0.07	−0.28*	0.20	0.05	0.13	0.13
Chao1	0.14	0.07	0.24	−0.11	−0.23	−0.22	0.29*	0.08	0.18	0.22
Bray-Curtis dissimilarity	0.42***	0.52***	−0.03	0.26*	0.08	0.38**	–0.03	−0.05	0.35**	0.48***
PNC	−0.07	−0.07	−0.14	0.27*	−0.25	−0.65***	0.03	−0.41***	0.39**	0.23*
βNTI	0.50***	0.53***	0.08	−0.22	0.14	0.12	0.02	−0.02	0.43***	0.39**
AWCD	0.46***	0.48***	0.09	−0.14	−0.22	−0.29*	0.35*	−0.11	n.a.	n.a.
SOC mineralization	0.36**	0.43**	−0.01	−0.02	−0.31	−0.27	0.32	−0.14	n.a.	n.a.

a Partial Mantel tests for the correlations between soil properties and the bacterial community composition (Bray-Curtis dissimilarity) and assembly processes (βNTI, the weighted beta nearest taxon index). Partial correlation tests were performed to determine the correlations between soil properties, the bacterial biomass, diversity (Shannon index and Chao1 richness), and the percentage of negative correlations (PNC) in the cooccurring networks, carbon metabolic activities, and soil organic carbon (SOC) mineralization. Soil properties included soil pH, total phosphorus (TP), SOC, total nitrogen (TN), total potassium (TK), soil moisture content (SMC), nitrate nitrogen (NO_3_−N), and ammonia nitrogen (NH_4_−N). Calculation of values representing Shannon index and Chao1 richness was performed on the basis of OTU tables rarified to the same sequencing depth. The microbial metabolic activities are reflected by the average well color development (AWCD). SOC mineralization was measured by analyzing cumulative CO_2_ emission in the microcosms. The significance of results of comparisons was tested based on 999 permutations. ***, *P *< 0.001; **, *P *< 0.01; *, *P *< 0.05; n.a., not analyzed. All analyses were conducted based on all 60 samples.

**FIG 5:**
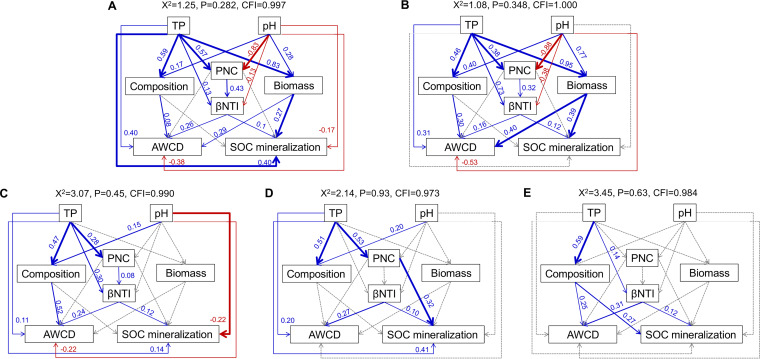
Structural equation modeling shows the direct and indirect effects of soil properties and the bacterial community on carbon metabolism and SOC mineralization in five soil depth layers, including 0 to 10 cm (A), 10 to 20 cm (B), 20 to 40 cm (C), 40 to 60 cm (D), and 60 to 80 cm (E). Blue lines indicate positive effects, and red lines indicate negative effects. The width of arrows indicates the strength of significant standardized path coefficients. Paths with nonsignificant coefficients are presented as gray lines. Soil properties include pH and total phosphorus (TP), while the bacterial community data include biomass, composition (Bray-Curtis dissimilarity), assembly processes (βNTI, the weighted beta nearest taxon index), and the cooccurring networks (PNC, the percentage of negative correlations). Carbon metabolic activities are reflected by the average well color development (AWCD). SOC mineralization is indicated by cumulative CO_2_−C.

Structural equation modeling (SEM) was developed to explore the potential direct and indirect effects of abiotic and biotic factors on carbon metabolism and SOC mineralization (Fig. 5). Overall, soil TP was positively linked to carbon metabolism in the 0-to-10 cm, 10-to-20 cm, and 20-to-40-cm layers, while pH showed a negative relationship (Fig. 5). Soil TP and pH were not only directly correlated with community assembly in the 0-to-10-cm and 10-to-20-cm layers but also indirectly related to community assembly via PNC. The bacterial biomass had significantly positive relationships with carbon metabolism in the 0-to-10-cm and 10-to-20-cm layers. In addition, the community composition (Bray-Curtis dissimilarity) and assembly (βNTI) exhibited positive relationships with carbon metabolism in the 0-to-10 cm, 10-to-20-cm, 20-to-40-cm, and 60-to-80-cm layers.

## DISCUSSION

### Stochastic and deterministic processes structure bacterial community assembly.

Uncovering microbial community assembly processes is a long-standing and extremely challenging task (1–3). Our results showed that the bacterial community assembly was governed by determinism and stochasticity, with the relative contribution of stochasticity increasing progressively in the deeper soils. Stochastic and deterministic processes represent two complementary mechanisms along a continuum of ecological forces that structure community assembly (3). The 0-to-10-cm layer was characterized by the strong variable selection (40%) of determinism (|βNTI| > 2) guiding community assembly with a relatively lower *m* value and higher β-null deviations (Fig. 2). This result may be attributed to the high selection forces involving environmental heterogeneity and biological interactions in topsoil. However, the bacterial communities in the subsoil (10 to 80 cm) with lower environmental heterogeneity were more extensively governed by stochastic processes, which may consist of ecological drift, probabilistic dispersal, and/or random births/deaths (26). Homogenizing dispersal was largely responsible for the bacterial community assembly in the 10-to-80-cm layer (43% to 91%), which was supported by the high value of *m* and low β-null deviations (Fig. 2). In this case, homogenizing dispersal greatly homogenized the bacterial community structure and caused low compositional turnover (2, 11). Collectively, the results suggested the importance of the deterministic and stochastic processes that complementarily and simultaneously governed the bacterial community across the soil profiles. The abundant groups in the bacterial communities were further parsed to better understand the taxon-specific selection and dispersal mechanisms (27–29). In our opinion, the type of ecological processes structuring the assembly of the bacterial phyla was depth dependent. We found that stochasticity was important for *Acidobacteria*, *Actinobacteria*, *Alphaproteobacteria*, *Chloroflexi*, and Deltaproteobacteria, with the decreased strength of variable selection but the increased homogenizing dispersal. Dispersal rates depend largely on species traits (flagella, cell size, metal resistance ability, etc.) and the activity status of bacterial taxa, ranging from being quite restricted to being extremely unrestricted (3). Homogenizing dispersal coupled with high migration rates can unify the species pool of the bacterial community and hence lead to low compositional variation (2, 11, 30).

### Environmental filtering and bacterial cooccurrence patterns drive community assembly.

Depth gradients, which are imposed by abiotic environment filtering, are a predominant driver of community assembly processes. Soil microbial community dissimilarities between the surface and subsurface soil at a single site were equal to or greater than those at multiple sites across hundreds of kilometers, indicating the powerful effect of filtering across the soil profile (31). The patterns in our study suggested that the directional shifts in the stochastic-deterministic balance were mirrored by the spatial variations in soil pH and TP in the 0-to-10-cm and 10-to-20-cm layers. It has long been recognized that soil properties are crucial determinants of soil bacterial community assembly (32, 33). For instance, soil pH has been identified as the key factor that mediates the relative influences of stochastic and deterministic processes of bacterial community assembly at a broad range of scales (7, 32). Manure fertilization supplies considerable quantities of organic P and effectively improves P-sorption capacity in acidic soils (33, 34). A large shift in TP is expected to cause strong ecological selection to promote the compositional turnover of soil bacterial communities. Nevertheless, the levels of strength of the selection imposed by pH and TP on bacterial community assembly differed across soil profiles. The variations in pH and TP were much lower in deep soil layers than in the other layers (see Fig. S1 in the supplemental material), suggesting that the soil environment became more homogeneous with increasing soil depth. As a consequence, the bacterial communities in the 20-to-80-cm layer should be less affected by environmental filtering, i.e., by deterministic processes.

The structural properties of networks allow comparisons between soil layers to understand how biotic cooccurrence patterns shape bacterial community assembly (18, 35). The low values of PNC in the subsoil networks (20 to 80 cm) indicated weak competition among the dominant phyla in the bacterial assemblages. Accordingly, decreased negative bacterial relationships were expected to decrease the relative influence of variable selection and to impose low compositional turnover in the bacterial community due to the homogenous environment. Intriguingly, the extent of the discrepancy between the obtained (*O*) and random (*R*) cooccurring frequencies reflected differences among microhabitats across the soil profile (14, 36). Structural analysis demonstrated that the stochastic pattern of intraphylum cooccurrence was prevalent in the dominant phyla *Acidobacteria* and *Chloroflexi* in the 40-to-60-cm and 60-to-80-cm layers (Fig. 4; see also Table S2 in the supplemental material), which is consistent with the results of βNTI. We extrapolated from the data the finding that homogeneous dispersal-based stochasticity dispersed its members broadly, restricting the intraphylum cooccurrence in the networks. Therefore, the measured *O*/*R* ratio may provide insights into the ecological mechanisms guiding bacterial community assembly. Future manipulative and experimental studies are required to directly characterize the mechanisms that produce patterns of community assembly.

### Coupling bacterial community assembly to carbon metabolism.

The microbial community is expected to influence microbial functions related to carbon metabolism (21–23). Our results showed that the biomass, composition, and assembly processes of the bacterial community exhibited significant contributions to carbon metabolism and SOC mineralization (Table 1). Consistent with many previous studies (37, 38), our study indicated that bacterial biomass was the most important factor mediating carbon metabolism and SOC mineralization. However, the significant relationship between microbial assembly processes and carbon metabolism remains poorly understood. Our results suggested that the levels of carbon metabolism and SOC mineralization were highest when selective pressures were maximized but dispersal was minimized. Variable selection would putatively favor a well-adapted community with respect to prevailing conditions, resulting in increased carbon metabolic capacity and SOC mineralization (25). In contrast, high homogeneous dispersal may increase the proportion of maladapted organisms in bacterial communities that are vulnerable to changes in local environmental conditions (4, 25). The greater niche breadth of the bacterial community in deeper soils suggested a relatively lower maximum fitness level for the individuals and lower biogeochemical function (39). If individuals are dispersed to a more extreme environment, then they may invest more energy in maintaining cell survival to obtain energy for growth and reproduction (25). Considering the trade-off between function and vulnerability in the bacterial community, the ability to adjust entire community rates of carbon metabolism and SOC mineralization would decrease in response to perturbation. Our results provide new evidence supporting the idea that bacterial community assembly may influence carbon metabolism in deep soil layers. Potential mechanisms of positive coupling between ecological assembly processes and SOC dynamics still need further experimental investigation.

### Conclusions.

In summary, we provided a framework to enable better understanding of the mechanisms governing the balance between stochastic and deterministic processes and to integration of community assembly with microbial carbon metabolism. Specifically, stochasticity and determinism formed the ends of a continuum along soil profiles, wherein determinism weakened while stochasticity strengthened as soil depth increased. The assembly of the two most dominant phyla, *Acidobacteria* and *Chloroflexi*, followed a trend similar to that of the entire bacterial community. Environmental filtering and cooccurrence patterns jointly influenced the stochastic/deterministic continuum of soil bacterial community assembly. Our study results suggest that bacterial community assembly is associated with carbon metabolism. Theoretical and experimental delineation of the potential linkages between community assembly and function across a broad range of ecosystems represents an interesting topic for future research.

## MATERIALS AND METHODS

### Experimental design.

A long-term manure experiment was performed at the Yingtan National Agro-Ecosystem Observation and Research Station (28°15′20′′N, 116°55′30′′E) of the Chinese Academy of Sciences, Jiangxi Province, China. This region is characterized as having a typical subtropical climate, mean annual temperature of 17.6°C, and mean annual precipitation of 1,795 mm. The soil is acidic loamy clay developed from Quaternary red clay and is classified as a Ferric Acrisol according to the Food and Agriculture Organization of the United Nations (FAO) classification system and as a Udic Ferralsol according to Chinese soil taxonomy. The field experiment was established in accordance with a randomized design with four pig manure rates: (i) no manure (M0); (ii) low manure rate with 150 kg N ha^−1^ per year (M1); (iii) high manure rate with 600 kg N ha^−1^ per year (M2); and (iv) high manure rate with 600 kg N ha^−1^ per year and lime applied at 3,000 kg Ca(OH)_2 _ha^−1^ every 3 years (M3). Three replicates of each treatment had been applied to 2 m-long, 2 m-wide, and 1.5 m-deep plots since 2002. The pig manure on a dry-matter basis contained total carbon of 397.5 g kg^−1^ and total nitrogen (TN) of 34.5 g kg^−1^. Rotations of monocropped maize (cultivar no. 11 from Denghai) was planted annually in April and harvested in July from 2002 to 2017. There were no tillage and management measures with the exception of weeding by hand.

### Soil sampling and physicochemical characteristics.

Soil samples in each plot were collected at a depth of 0 to 80 cm in late July 2017. Within each plot, five soil cores (6 cm in diameter) free from maize roots were collected at random locations and partitioned into five depth intervals: 0 to 10 cm, 10 to 20 cm, 20 to 40 cm, 40 to 60 cm, and 60 to 80 cm. A total of 60 soil samples were collected, including 4 (fertilizer treatments) × 5 (soil layers) × 3 (replicates). Fresh samples were chilled on ice immediately following collection in the field and then transported in a cooler to the laboratory, where they were sieved (4-mm pore size) to remove roots and rocks. Then, the soil samples were gently broken along natural fracture planes to a size of <2 mm to measure the soil physicochemical properties and the bacterial community.

Soil physicochemical properties were then detected according to methods described in a handbook of soil analysis (40). Soil pH was determined using a glass electrode in a soil/water ratio of 1:2.5 (wt/vol). Soil organic carbon (SOC) was titrated against 0.5 M ferrous iron solution after it had been digested with 0.8 M K_2_Cr_2_O_4_ and concentrated H_2_SO_4_ (1:1 [vol/vol]) at 150°C for 30 min. Total nitrogen (TN) was measured as Kjeldahl N. Briefly, the soil sample was heated and boiled with concentrated H_2_SO_4_. The solution was then absorbed by the use of a 2% boric acid solution and titrated against 0.1 M sulfuric acid. Total phosphorus (TP) was extracted with HF−HClO_4_ and sodium bicarbonate, and TP levels were then determined by the molybdenum blue method using an UV spectrophotometer at 700 nm. Total potassium (TK) levels were determined using flame emission spectrometry after the soil had been digested in concentrated HF−HClO_4_ (2:1[vol/vol]). NH_4_−N and NO_3_−N were extracted with 2 M KCl and detected on a continuous flow analyzer (Skalar, Breda, Netherlands). SMC levels were measured by determination of the weight loss after 48 h of drying at 70°C.

### Carbon metabolic profiles and SOC mineralization.

The capability of soil microbial communities to utilize a variety of carbon sources was measured with Biolog EcoPlates (Biolog Inc., USA) (41). The Biolog EcoPlates system consisted of 31 different carbon sources plus a blank well in three replications. The carbon sources were subdivided into six group substrates, including carbohydrates, carboxylic acids, amino acids, polymers, phenolic acids, and amines (42). Soil microorganisms were extracted in accordance with the procedure described here. First, 5 g of soil (dry weight equivalent) was added to 45 ml of sterile 0.85% (wt/vol) saline solution. The mixture was then shaken for 30 min at 90 rpm on an orbital shaker and left to stand for 2 h. Afterward, 1 ml of the supernatant was diluted to 20 ml with sterilized saline solution. Each well of the Biolog EcoPlates was inoculated with 200 μl of the mixed suspension, after which the plates were incubated at 25°C in the dark for 7 days. The utilization rate of the carbon sources was determined by the reduction in tetrazolium violet redox dye, which changes from colorless to purple if added microorganisms utilize the substrate. The average well color development (AWCD), which indicates the carbon utilization, was calculated by taking absorbance measurements at 590 nm every 24 h. For the posterior analysis, absorbance at a single time point (96 h) was used, when the asymptote was reached. Measurements of optical density at 590 nm (OD_590_) from each well were corrected by subtracting the control (blank well) values from the values representing each plate well.

The SOC mineralization was measured by the cumulative CO_2_ efflux from soils using the alkali-absorption method. Briefly, 50-g (dry weight) soil samples were adjusted to 65% field capacity and preincubated in 250-ml sealed flasks to activate the soil microbial community for 7 days at 25°C. The content of CO_2_ trapped in 0.5 mol liter^−1^ sodium hydroxide (NaOH) was determined by titration at days 1, 3, 5, 7, 14, 21, 28, 42, 56, 70, 85, and 100. After each sampling, the incubation flasks were left open for 1 h in the surrounding air to reach the ambient O_2_ level and were refilled with fresh NaOH solution. Each treatment was replicated three times, and three control vials (silica sand without soil) were set to detect the concentration of background CO_2_. Soil moisture was maintained by adding distilled water throughout the incubation experiment. The SOC mineralization level mainly represents the decomposition of active SOC in the incubation experiment. We used a first-order kinetic equation, *C_t_* = *C*_0_ [1 − exp(−*kt*)], to fit the SOC mineralization processes, where *C_t_* represents the cumulative amount of CO_2_−C mineralized from the SOC at a certain incubation time (mg kg^−1^), *C*_0_ represents the potentially mineralized C (mg kg^−1^) and, mainly, the active carbon pool, *k* is the mineralization rate constant (day^−1^), and *t* is the incubation time (in days).

### Illumina sequencing and quantitative PCR (qPCR).

DNA was extracted from 0.5 g of fresh soil via the use of a MoBio Power Soil DNA extraction kit (MoBio Laboratories, Inc., CA, USA) in accordance with the manufacturer’s instructions. The quality and quantity of DNA were checked using a NanoDrop spectrophotometer (NanoDrop Technologies, Wilmington, DE, USA). The hypervariable V4−V5 region of bacterial 16S rRNA gene was amplified using a set of universal primer pairs, 515F (5′-GTGCCAGCMGCCGCGGTAA-3′) and 907R (5′-CCGTCAATTCCTTTGAGTTT-3′), for the Illumina sequencing (43). Both the forward and reverse primers were tagged with an adapter and linker sequence, and barcode oligonucleotides that were 8 bp in length were added to the reverse primer to distinguish the 16S rRNA amplicons that originated from different soil samples. Reaction mixtures (20 μl) contained 4 μl of 5× FastPfu buffer, 0.25 μl of each primer (10 μM), 2 μl of 2.5 mM deoxynucleoside triphosphates (dNTPs), 10 ng template DNA, and 0.4 μl FastPfu polymerase. The PCR protocol was as follows: an initial predenaturation at 95°C for 5 min followed by 28 cycles of 30 s at 94°C, 30 s at 55°C, and 45 s at 72°C and a final extension at 72°C for 10 min. All amplicons were cleaned and pooled in equimolar concentrations in a single tube, after which they were subjected to library preparation, cluster generation, and 300-bp paired-end sequencing on an Illumina MiSeq platform (Illumina Inc., San Diego, CA).

The raw sequences were quality screened and trimmed using the Quantitative Insights into Microbial Ecology (QIIME) pipeline (v1.9.1) (44). Sequences that fully matched the barcodes were selected and distributed into separate files for the bacterial 16S rRNA gene. Additional sequence processing, including quality trimming, demultiplexing, and taxonomic assignments, was performed. QIIME quality trimming was performed in accordance with the following criteria: (i) sequence reads were truncated before three consecutive low-quality bases and reevaluated for length, (ii) no ambiguous bases were allowed, and (iii) the minimum sequence length was 392 bp after trimming. The assembled reads were processed for *de novo* chimera detection conducted with UCHIME v5.1 (45). The remaining sequences were additionally screened for frame shifts via the use of HMM-FRAME (46). Thereafter, the 16S rRNA gene sequence was subjected to a similarity search on a one-by-one basis against sequences within the Ribosomal Database Project (RDP). Finally, the sequence reads from each sample were clustered to provide similarity-based operational taxonomic units (OTUs) that had 97% identity cutoff values (47). A total of 4,910 bacterial OTUs were generated after rarefication to 20,515 sequences per sample (based on the sample with the minimum numbers of reads) using the “*multiple_rarefractions_even_depth.py*” command. The alignment was filtered to remove common gaps using the “*align_seqs.py*” command, and a phylogenetic tree was constructed *de novo* using FastTree (48). Values representing alpha diversity (calculated using the “*alpha_diversity.py*” command) and Bray-Curtis dissimilarity of soil bacterial communities were calculated in a principal-coordinate analysis (using R package “vegan”).

Bacterial biomass was quantified by determination of the copy number of the 16S rRNA gene using qPCR and a Bio-Rad CFX96 Touch real-time PCR detection system. The high-quality DNA was amplified using the primers described above for the preparation of standard plasmids (43). The qPCR products were electrophoresed on a 1% agarose gel containing ethidium bromide and visualized using a gel image processing system (Tanon-1600; Tanon Science & Technology Co., Ltd., Shanghai, China). The qPCR products were cloned using a pUC-T TA cloning kit (CoWin Biosciences, Beijing, China) and then were transformed into Escherichia coli DH5a competent cells. The plasmids of the positive clones containing the 16S rRNA gene fragment were used as plasmid DNA standards. Standard curves for the bacterial community were observed using a dilution series (10^2^ to 10^8^ copies) of plasmid DNA (49). Calibration curves were generated with Sequence Detection system software according to the qPCR results determined with the plasmid DNA standards and dilutions. Reaction mixtures (20 μl) contained 1 μl DNA template (1 to 10 ng), 10 μl 2 × SYBR Premix *Ex Taq*, and a 0.5 μM concentration of each primer. All qPCR assays were run with 3 min initial denaturation at 95°C followed by 40 cycles (with plate reading) of 30 s at 95°C and 45 s at 60°C and by a final melt-curve step from 72 to 95°C. No-template controls were included with each qPCR run. The qPCRs were run in triplicate, and amplification efficiencies of >97% were obtained with *R*^2^ values of >0.99.

### Ecological network construction.

To describe the complex cooccurrence pattern of bacterial networks, we constructed a correlation matrix by calculating multiple correlations and similarities with Co-occurrence Network (CoNet) inference (50). Twelve samples (4 fertilization treatments × 3 replicates) in each soil layer were used to construct the bacterial networks. In total, five networks were constructed from each of five soil layers. The OTUs detected in more than three-fourths of the soil samples at the same depth were kept for the network construction. We used an ensemble approach that combined four measurements, including Pearson and Spearman correlations and Bray-Curtis and Kullback-Leibler dissimilarities. Weighted voting with Brown *P* values (51) was used for the four measurements, as this method accounts for the dependency among measures. A valid cooccurrence was considered a statistically robust correlation between species when the correlation coefficient (*r*) was greater than 0.6 or less than −0.6 and the *P* value was <0.01. Those *P* values that were <0.01 were adjusted by a testing correction using the Benjamini-Hochberg procedure to reduce the chances of obtaining false-positive results. Correlation networks were visualized via Gephi software (52). The calculated topological characteristics of the bacterial networks included average path length, graph density, network diameter, average clustering coefficient, average degree, and modularity. The discrepancy between the observed (*O*) and random (*R*) cooccurring incidences was used to display nonrandom assembly patterns in the bacterial communities (14). Briefly, *O*% was calculated as the relative percentage of the number of observed edges (*Eo*) between two taxa divided by total number of edges (*E*) in the network, while the random coexcluding incidence (*R*%) was theoretically calculated by considering the frequencies of two taxa [*n* (N1) and *n* (N2)] and random associations. The ratio of *O*% to *R*%, that is, the *O*/*R* ratio, was used as a measure of nonrandom species-species associations between two different taxa. When the *O*/*R* ratio is >1, the network tends to determinism. When the *O*/*R* ratio is <1, the network tends to stochasticity.

### Community assembly processes and habitat niche breadth.

The weighted β nearest taxon index (βNTI) and Bray-Curtis-based Raup-Crick (RC_bray_) values were calculated via a null model methodology to differentiate the ecological processes that regulate bacterial community assembly (2, 11). The βNTI was quantified by determination of the standard deviation between an observed level and the null distribution of the mean nearest taxon distance metric (βMNTD). The pairwise phylogenetic turnover between communities was calculated as βMNTD to infer the community assembly processes via the “comdistnt” function of the picante package (53). The RC_bray_ data were calculated by the determination of the deviation between the empirically observed Bray-Curtis data and the null distribution via the use of the vegan package (54), with the values seen after standardization ranging between −1 and +1 (2). Specifically, deterministic processes were associated with variable selection (βNTI greater than 2) and homogeneous selection (βNTI less than −2), and stochastic processes were associated with homogenizing dispersal (|βNTI| greater than 2 and RC_bray_ less than −0.95) and dispersal limitation (|βNTI| greater than 2 and RC_bray_ greater than 0.95). Undominated processes that were not dominant (|βNTI| greater than 2 and |RC_bray_| greater than 0.95) indicated a situation where composition turnover was not dominated by any single process as described above. Specifically, “homogeneous selection” refers to the primary cause of the low rate of compositional turnover caused by the consistent selective pressure that results from consistent environmental conditions; “variable selection” refers to the primary cause of high compositional turnover caused by a shift in selective pressure that results from a shift in environmental conditions; “homogenizing dispersal” refers to the high dispersal rate between the communities in a given pair that is the primary cause of low compositional turnover; “dispersal limitation” refers to the high turnover in composition that is primarily due to a low rate of dispersal-enabling community composition; and “undominated” refers to the compositional differences between communities that are due to a mixture of stochastic organismal movements and stochastic birth-death events (11).

The abundance-based β-null model was used to distinguish deterministic from stochastic processes by evaluating the deviation between the observed β-diversity and null-expected β-diversity of a randomly assembled pair of communities (8, 55). The higher the null deviation value of a microbial community, the greater the effect of the deterministic processes by which they will be influenced. Custom R scripts for β-null model fitting written by Tucker et al. (8) were modified to include the weighted UniFrac β-null model described previously by Lee et al. (55). We applied two distance metrics, including Bray-Curtis and weighted UniFrac, for the analysis of microbial communities. We calculated the pairwise β-diversity between the samples in 999 randomly assembled pairs to produce a distribution of null β-diversity.

The Sloan neutral model was used to estimate the importance of the effects of stochastic processes on community assembly (9). This model predicts that less-abundant taxa would be lost due to ecological drift, while more-abundant taxa are more likely to be dispersed by chance. Migration rate (*m*) data were calculated by analysis of observed OTU distributions and mean relative abundances. Higher *m* values indicate that microbial communities are less limited by dispersal. This analysis was performed using nonlinear least-squares fitting and the minpack.lm package in R (56). Calculation of 95% confidence intervals (CIs) for the model predictions was conducted using the Wilson score interval in the Hmisc package in R (57). Niche breadth was calculated using Levins’ niche breadth index (*B*) equation (58), Bj=1∑i=1NPij2, where *B_j_* represents the habitat niche breadth of OTU *j* in a metacommunity, *N* is the total number of communities of each metacommunity, and *P_ij_* is the proportion of OTU *j* in community *i*. A high *B* value indicates that the OTU occurs widely and evenly along a wide range of locations, representing wide habitat niche breadth. The *B* value representing the community level (*B*_com_) was calculated as the average of *B* values from all taxa occurring in one community. The microbial group with a wider niche breadth is thought to be more metabolically flexible at the community level.

### Statistical analyses.

Two-way PERMANOVA was used to estimate the effect of fertilization treatments and soil depth on soil properties, the bacterial community, carbon metabolic profiles, and SOC mineralization using SPSS 22.0 software (SPSS, Chicago, IL, USA). One-way analysis of variance (ANOVA) was performed to determine the statistically significant differences in soil properties, bacterial biomass, diversity, βNTI, AWCD, SOC mineralization, niche breadth, and β-null deviation value, based on the data that followed a normal distribution and had the same variances (59), along with the use of Turkey’s test for multiple comparisons (*P < *0.05). Variance-covariance matrix data based on all soil properties were calculated to indicate the overall variation in soil properties. A canonical principal-coordinate analysis (PCoA) was performed to estimate the influence of soil depth on the Bray-Curtis dissimilarities of bacterial community composition (60). We used “capscale” and “permutest” permutation-based testing functions for PCoA and for calculating significance values, respectively. Partial Mantel tests were performed to determine the correlations between soil properties and the bacterial community composition (Bray-Curtis dissimilarity) and βNTI. Partial correlation tests were performed to determine the correlations between soil properties, the bacterial biomass, and diversity (Shannon index and Chao1 richness) and PNC, carbon metabolism, and SOC mineralization.

The random forest tool was used to quantitatively illustrate the important predictors of carbon metabolic capacity and SOC mineralization corresponding to soil properties and the bacterial community. The soil property data included soil pH, SOC, TN, TP, TK, NH_4_–N, NO_3_–N, and SMC, while the bacterial community data included biomass, composition (Bray-Curtis dissimilarity), diversity (Shannon index), βNTI, and PNC. The total 60 samples were randomly divided into two parts with about 2/3 used for the training data set and the remaining samples for the “out-of-bag” data set (61). The importance of each factor was evaluated by analysis of the increase in the mean square error between the observed and predicted values seen when the predictor was randomly permuted (62). The accuracy of the importance results was measured for each tree and then averaged across the forest with 500 trees (63). Structural equation modeling (SEM) was applied to determine the direct and indirect contributions of abiotic and biotic variables to the bacterial community assembly and carbon metabolism data. The first step in SEM required establishing an *a priori* model based on the known effects of variables on the bacterial community assembly and carbon metabolic capacity. We excluded the predictors of poor fitting to the model and then established a unified structural equation modeling the data from each soil layer. SEM analysis was conducted via the robust maximum likelihood evaluation method using AMOS 20.0. The SEM fitness was examined on the basis of a nonsignificant chi-square test (*P > *0.05), the goodness-of-fit index, and the root mean square error of approximation (64).

All statistical analyses were performed in R (v3.5.1; http://www.r-project.org/), using the “picante” (53), “vegan” (54), “minpack.lm” (56), “hmisc” (57), “randomForest” (65), “A3” (66), “rfPermute” (67), “stats” (68), and “spaa” (69) packages.

### Data availability.

The sequences of the 16S rRNA gene were deposited in the Sequence Read Archive (SRA) at NCBI under accession number SRP151282. All data needed to evaluate the conclusions in the paper are present in the paper and/or the supplemental material. Additional data related to this paper may be requested from us.
