# Supplementary figures and images for: Coupling Bacterial Community Assembly to Microbial Metabolism across Soil Profiles
Source: mSystems. 2020 Jun 9;5(3):e00298-20. doi: 10.1128/mSystems.00298-20 (PMC7289589; doi:10.1128/mSystems.00298-20)

0-10 cm    10-20 cm    20-40 cm    40-60 cm    60-80 cm

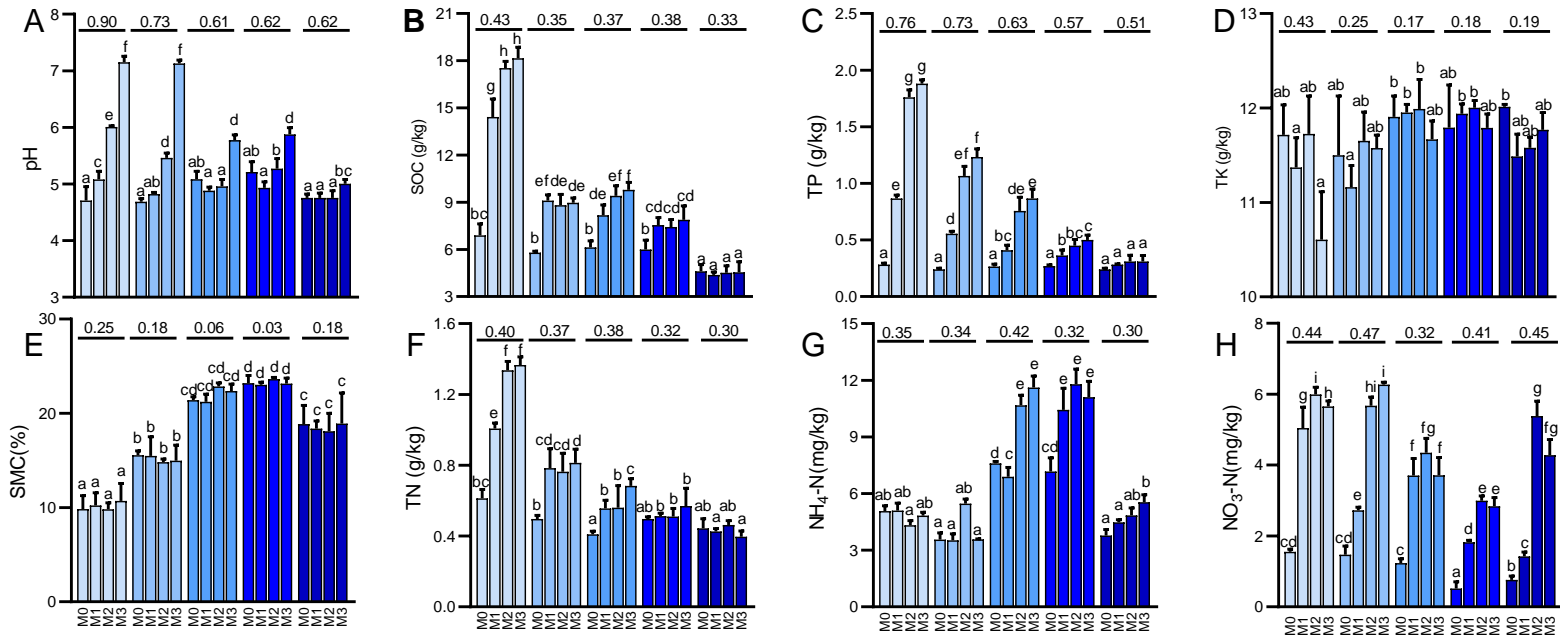

Supplement: FIG S1 [file mSystems.00298-20-sf001.pdf]

0-10 cm 10-20 cm 20-40 cm 40-60 cm 60-80 cm

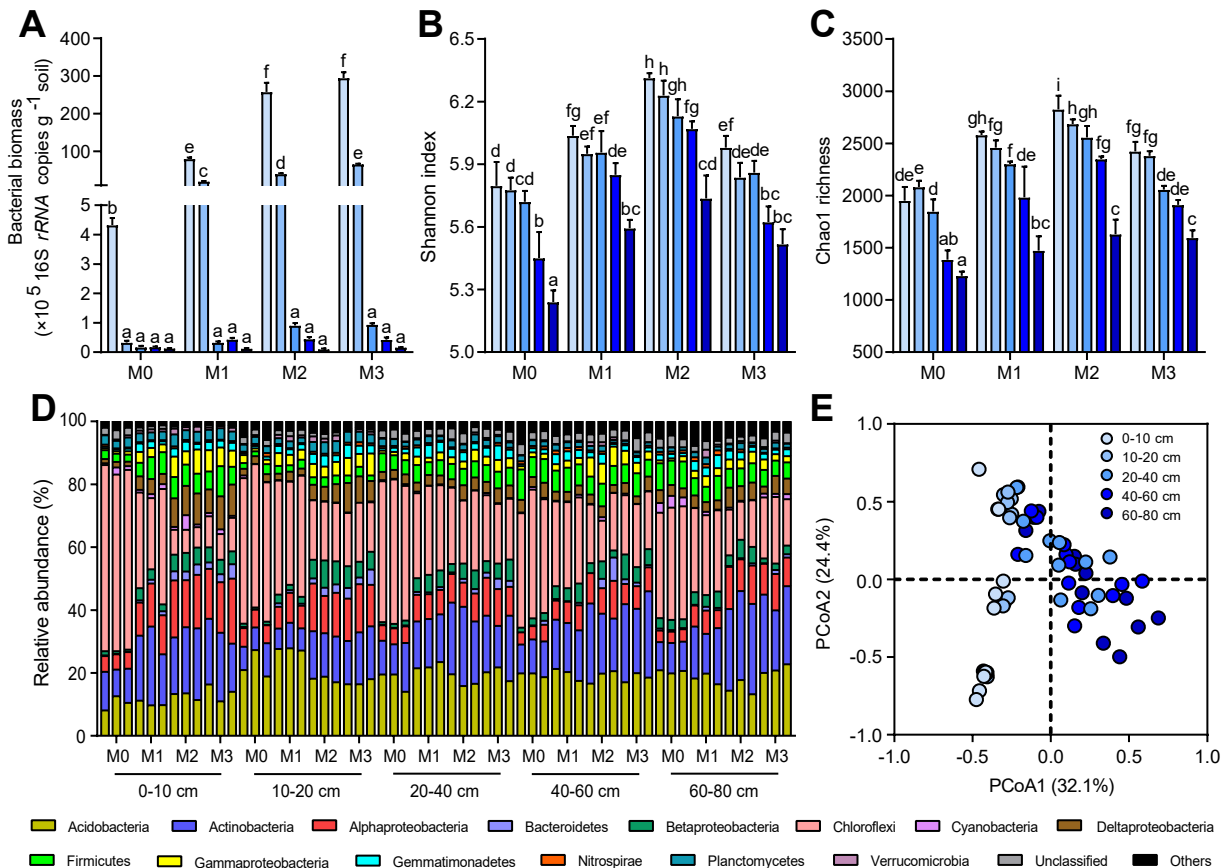

Supplement: FIG S2 [file mSystems.00298-20-sf002.pdf]

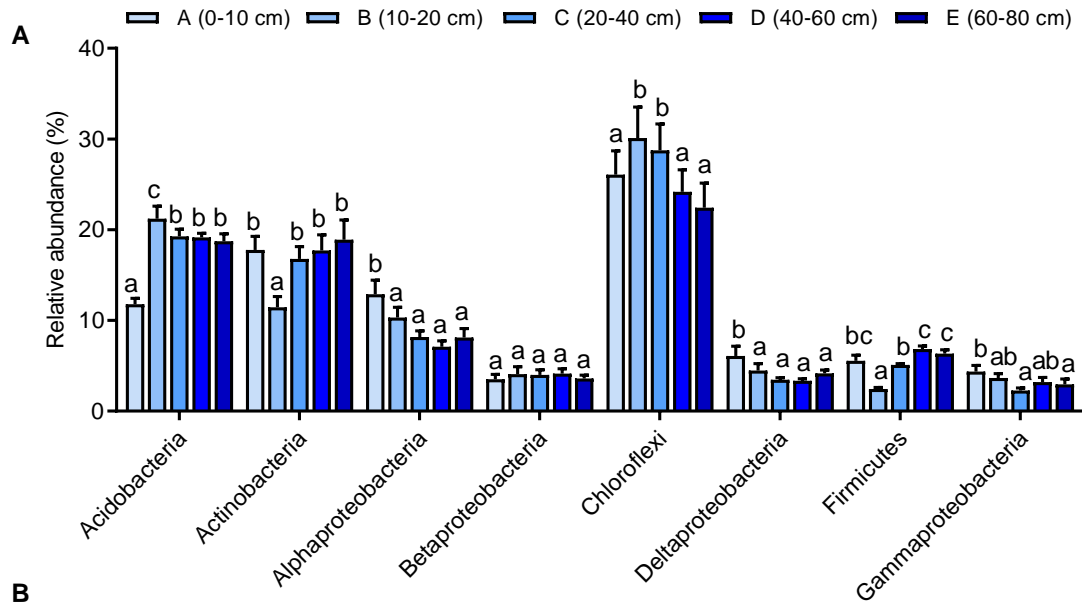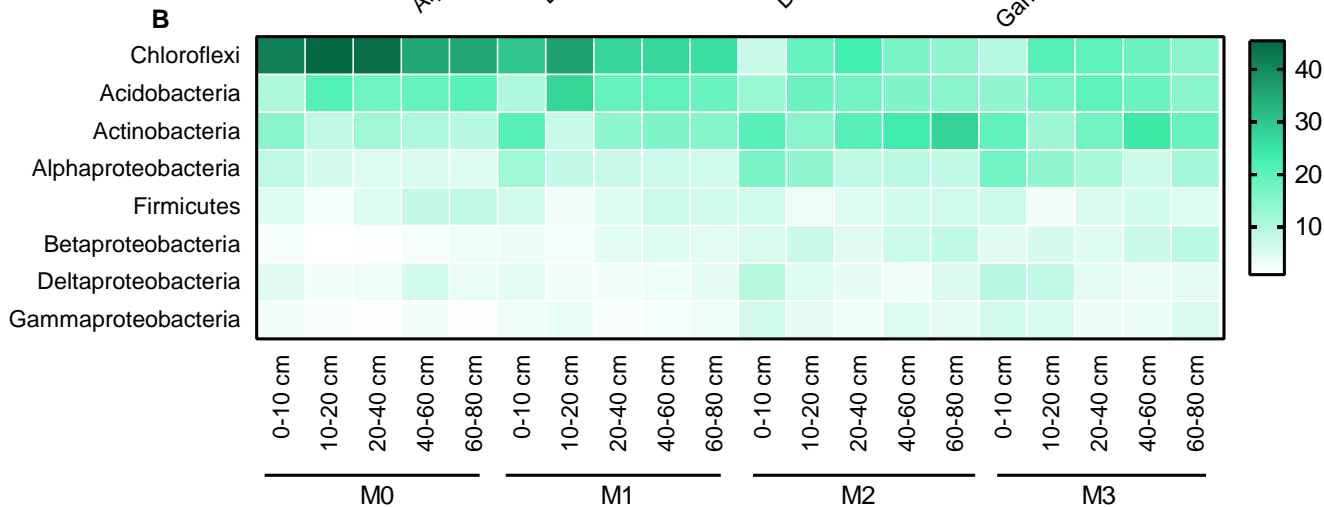

Supplement: FIG S3 [file mSystems.00298-20-sf003.pdf]

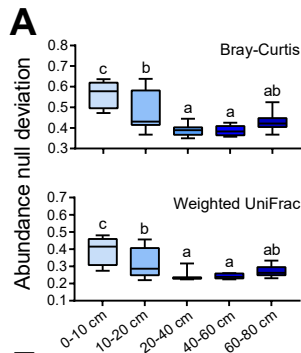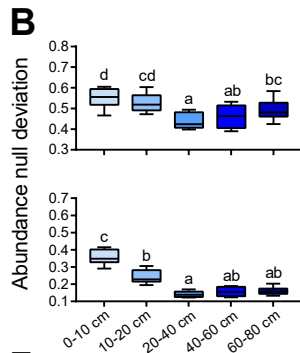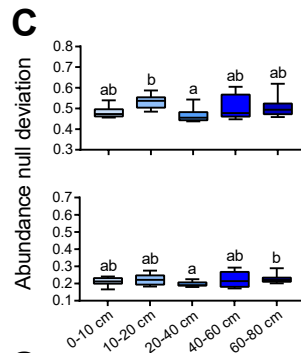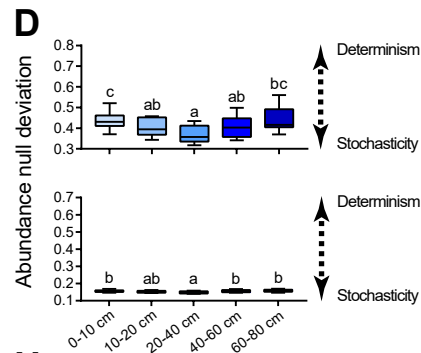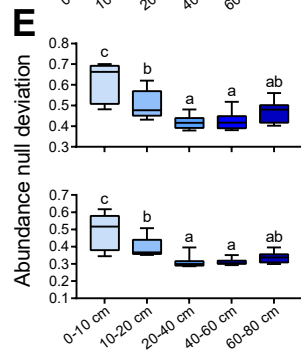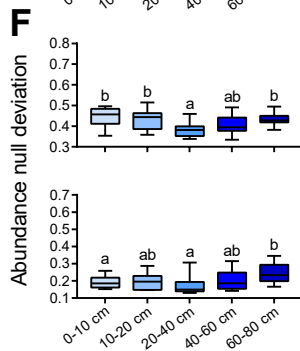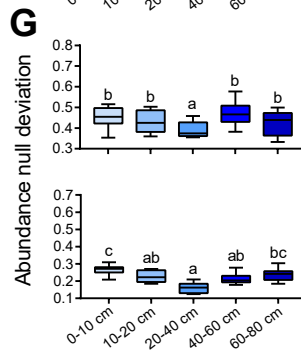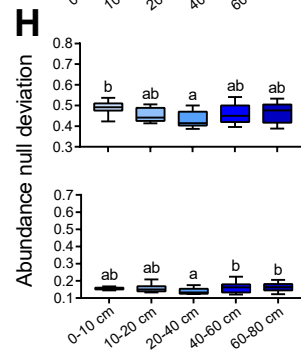

Supplement: FIG S5 [file mSystems.00298-20-sf005.pdf]

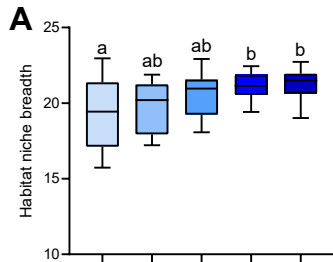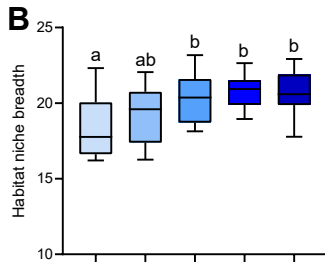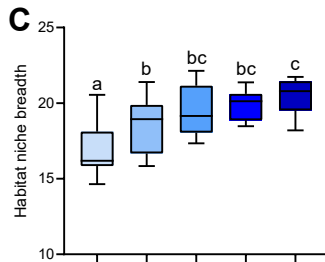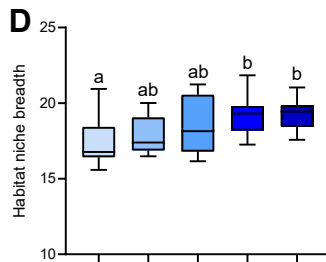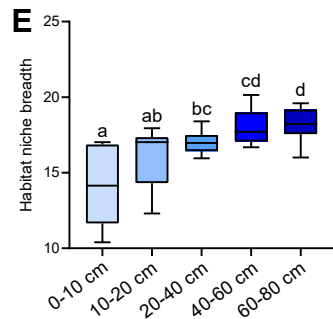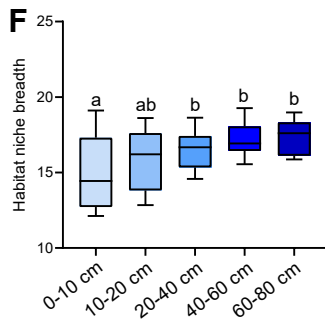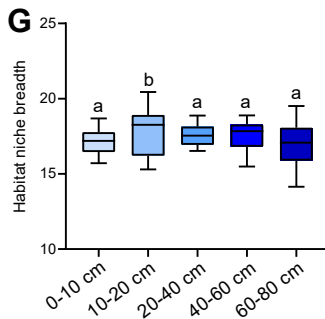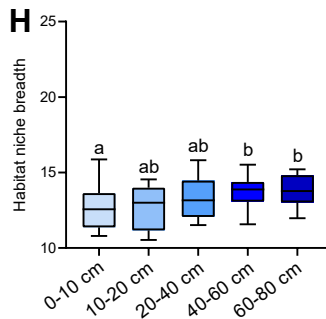

Supplement: FIG S6 [file mSystems.00298-20-sf006.pdf]
