# Supplementary material for: Coupling Bacterial Community Assembly to Microbial Metabolism across Soil Profiles
Source: mSystems. 2020 Jun 9;5(3):e00298-20. doi: 10.1128/mSystems.00298-20 (PMC7289589; doi:10.1128/mSystems.00298-20)
Supplement: TABLE S1 [file mSystems.00298-20-st001.pdf]

Table S1 Two-way PERMANOVA showing the effects of soil depth and fertilization treatments on soil properties, the bacterial community, carbon metabolism and SOC mineralization[a].

	Depth			Fertilization			Depth × Fertilization		
	Sums of Sqs	F	R^2	Sums of Sqs	F	R^2	Sums of Sqs	F	R^2
**Soil properties**	37.406	52.22	0.35***	27.022	23.84	0.16***	6.740	16.35	0.11***
TP	2.086	120.00	0.36***	2.036	120.64	0.36***	0.996	40.72	0.12***
pH	0.613	23.76	0.13***	1.665	67.68	0.38***	0.814	30.44	0.17***
SOC	2.186	95.09	0.51***	0.535	22.69	0.12***	0.395	11.39	0.06***
TN	2.212	137.80	0.57***	0.479	26.98	0.11***	0.485	21.07	0.09***
TK	0.221	8.71	0.13**	0.054	2.07	0.03	0.035	1.56	0.02
SMC	3.122	65.97	0.54***	0.003	0.03	0.00	0.000	0.04	0.00
NO_3-N	0.051	2.96	0.04	0.130	3.25	0.05*	0.151	6.07	0.09**
NH_4-N	0.241	2.61	0.04	0.000	0.32	0.00	0.004	0.08	0.00
Biomass	0.936	38.67	0.30***	0.095	12.93	0.10***	0.156	21.58	0.17***

Shannon	0.466	14.93	0.19***	0.011	0.36	0.01	0.170	5.48	0.07*
Chao1	0.876	19.69	0.25***	0.040	3.37	0.04	0.152	0.51	0.01
Bray-Curtis dissimilarity	1.976	15.96	0.16***	2.897	23.40	0.24***	0.399	3.23	0.03*
PNC	2.129	41.63	0.38***	0.003	0.32	0.01	0.578	11.29	0.10**
βNTI	72.324	106.59	0.59***	4.601	9.25	0.11***	7.034	10.37	0.06*
AWCD	1.713	56.67	0.40***	0.843	27.89	0.19***	0.383	13.83	0.09**
SOC mineralization	1.663	149.93	0.44***	1.034	110.65	0.32***	0.101	0.165	0.08*

a. Soil properties include pH, total phosphorus (TP), soil organic carbon (SOC), total nitrogen (TN), total potassium (TK), soil moisture content (SMC), nitrate nitrogen (NO_3–N) and ammonia nitrogen (NH_4–N). The bacterial community includes biomass, diversity (Shannon index and Chao1 richness), composition (Bray-Curtis dissimilarity), the bacterial assembly processes (βNTI, the weighted beta nearest taxon index), and the percentage of negative correlations (PNC) in the co-occurring networks. Calculation of by Shannon index and Chao1 richness is based on OTU tables rarified to the same sequencing depth. The microbial metabolic activities are reflected by the average well color development (AWCD). SOC mineralization is measured by cumulative CO_2 emission in the microcosms. *** $P < 0.001$, ** $P < 0.01$, * $P < 0.05$.
