# Supplementary material for: Coupling Bacterial Community Assembly to Microbial Metabolism across Soil Profiles
Source: mSystems. 2020 Jun 9;5(3):e00298-20. doi: 10.1128/mSystems.00298-20 (PMC7289589; doi:10.1128/mSystems.00298-20)
Supplement: FIG S4 [file mSystems.00298-20-sf004.pdf]

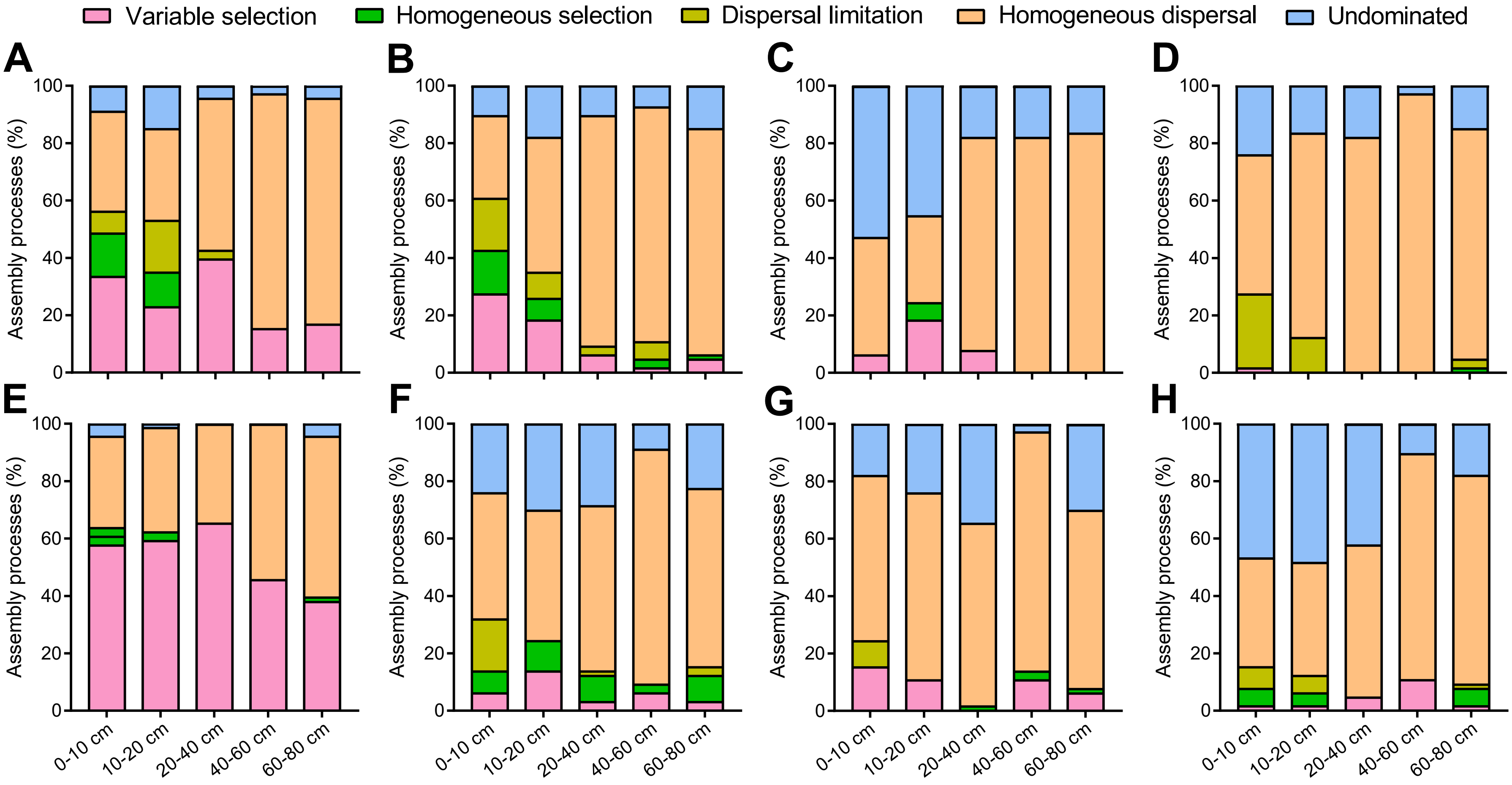

Variable selection
Homogeneous selection
Dispersal limitation
Homogeneous dispersal
Undominated
A
B
C
D
E
F
G
H
Assembly processes (%)
0
20
40
60
80
100
0-10 cm
10-20 cm
20-40 cm
40-60 cm
60-80 cm
