# Supplementary material for: Coupling Bacterial Community Assembly to Microbial Metabolism across Soil Profiles
Source: mSystems. 2020 Jun 9;5(3):e00298-20. doi: 10.1128/mSystems.00298-20 (PMC7289589; doi:10.1128/mSystems.00298-20)
Supplement: TABLE S2 [file mSystems.00298-20-st002.pdf]

Table S2 Parameters and fits of neutral models consider the influence of drift with the influence of dispersal across five soil profiles.

	Soil profiles	m[a]	m.ci	m.mle	maxLL	Rsqr	RMSE	AIC	BIC	Richness	%AbovePred	%BelowPred
All OTUs	0-10 cm	0.23	0.01	0.23	–893.00	0.48	0.20	–1781.99	–1769.33	4142.00	0.08	0.10
	10-20 cm	0.25	0.02	0.25	–1100.86	0.51	0.19	–2197.72	–2185.07	4126.00	0.07	0.09
	20-40 cm	0.36	0.02	0.36	–1287.94	0.64	0.18	–2571.88	–2559.21	4156.00	0.10	0.07
	40-60 cm	0.30	0.02	0.30	–1527.59	0.71	0.16	–3051.17	–3038.73	3859.00	0.08	0.06
	60-80 cm	0.35	0.01	0.35	–1447.57	0.63	0.17	–2891.14	–2878.63	3715.00	0.07	0.07
Acidobacteria	0-10 cm	0.17	0.03	0.17	–58.77	0.40	0.20	–113.54	–105.96	327.00	0.13	0.13
	10-20 cm	0.16	0.03	0.16	–84.12	0.54	0.19	–164.25	–156.64	331.00	0.13	0.11
	20-40 cm	0.25	0.05	0.25	–66.50	0.60	0.20	–128.99	–121.30	345.00	0.21	0.12
	40-60 cm	0.21	0.04	0.21	–106.63	0.72	0.17	–209.25	–201.71	331.00	0.20	0.09
	60-80 cm	0.25	0.03	0.25	–105.22	0.70	0.18	–206.44	–198.83	320.00	0.14	0.10
Actinobacteria	0-10 cm	0.24	0.05	0.24	–78.86	0.55	0.19	–153.72	–146.08	337.00	0.11	0.13

	10-20 cm	0.28	0.05	0.28	–99.93	0.59	0.18	–195.87	–188.17	347.00	0.12	0.10
	20-40 cm	0.42	0.08	0.42	–126.05	0.73	0.17	–248.11	–240.44	342.00	0.14	0.07
	40-60 cm	0.43	0.07	0.43	–153.56	0.78	0.15	–303.12	–295.54	343.00	0.11	0.05
	60-80 cm	0.44	0.04	0.44	–137.69	0.73	0.16	–271.39	–263.71	328.00	0.11	0.08
Alphaproteobacteria	0-10 cm	0.36	0.06	0.36	–128.43	0.60	0.17	–252.85	–244.97	381.00	0.09	0.06
	10-20 cm	0.33	0.06	0.33	–134.83	0.68	0.17	–265.67	–257.92	356.00	0.10	0.08
	20-40 cm	0.46	0.08	0.46	–145.98	0.73	0.16	–287.95	–280.21	354.00	0.12	0.08
	40-60 cm	0.36	0.06	0.36	–163.61	0.76	0.15	–323.23	–315.59	351.00	0.07	0.06
	60-80 cm	0.39	0.03	0.39	–133.21	0.66	0.17	–262.42	–254.69	336.00	0.07	0.07
Betaproteobacteria	0-10 cm	0.19	0.07	0.19	–22.30	0.62	0.19	–40.59	–35.68	86.00	0.15	0.09
	10-20 cm	0.20	0.05	0.20	–37.03	0.75	0.16	–70.05	–65.12	87.00	0.09	0.06
	20-40 cm	0.14	0.04	0.14	–20.22	0.64	0.19	–36.43	–31.67	80.00	0.16	0.08
	40-60 cm	0.21	0.06	0.21	–40.78	0.81	0.14	–77.56	–72.93	75.00	0.11	0.05

	60-80 cm	0.19	0.02	0.19	–37.35	0.78	0.15	–70.69	–66.11	73.00	0.14	0.03
	0-10 cm	0.10	0.01	0.10	–152.49	0.55	0.21	–300.98	–291.19	991.00	0.06	0.11
	10-20 cm	0.19	0.02	0.19	–222.09	0.41	0.19	–440.18	–430.36	1003.00	0.07	0.10
Chloroflexi	20-40 cm	0.37	0.04	0.37	–284.85	0.63	0.18	–565.70	–555.83	1029.00	0.13	0.07
	40-60 cm	0.35	0.04	0.35	–356.70	0.70	0.16	–709.40	–699.73	937.00	0.08	0.07
	60-80 cm	0.35	0.02	0.35	–353.98	0.59	0.17	–703.95	–694.27	928.00	0.06	0.06
	0-10 cm	0.43	0.07	0.43	–123.62	0.61	0.16	–243.24	–235.79	306.00	0.07	0.06
	10-20 cm	0.38	0.08	0.38	–90.99	0.54	0.18	–177.99	–170.70	283.00	0.08	0.07
Deltaproteobacteria	20-40 cm	0.55	0.11	0.55	–113.86	0.64	0.16	–223.71	–216.47	276.00	0.08	0.05
	40-60 cm	0.45	0.08	0.45	–150.26	0.76	0.13	–296.52	–289.44	260.00	0.04	0.04
	60-80 cm	0.36	0.03	0.36	–149.13	0.68	0.14	–294.26	–287.14	254.00	0.02	0.05
Firmicutes	0-10 cm	0.66	0.23	0.66	–36.5	0.62	0.18	–69.01	–63.38	139	0.05	0.07
	10-20 cm	0.55	0.18	0.55	–58.21	0.75	0.15	–112.41	–106.8	122	0.02	0.06

	20-40 cm	0.60	0.17	0.60	–56.82	0.77	0.16	–109.63	–103.91	129	0.04	0.07
	40-60 cm	0.54	0.09	0.54	–41.9	0.74	0.17	–79.79	–74.2	121	0.07	0.06
	60-80 cm	0.58	0.05	0.58	–57.35	0.72	0.16	–110.7	–104.83	123	0.05	0.08
	0-10 cm	0.20	0.06	0.20	–34.50	0.43	0.19	–65.01	–58.99	150.00	0.07	0.10
	10-20 cm	0.21	0.06	0.21	–39.17	0.47	0.19	–74.33	–68.34	148.00	0.07	0.09
Gammaproteobacteria	20-40 cm	0.20	0.05	0.20	–66.13	0.65	0.15	–128.25	–122.29	146.00	0.08	0.05
	40-60 cm	0.16	0.04	0.16	–53.40	0.67	0.16	–102.79	–97.04	139.00	0.06	0.07
	60-80 cm	0.14	0.03	0.14	–51.10	0.63	0.17	–98.21	–92.34	131.00	0.09	0.07

a. The Sloan neutral model parameters: *m* is a single free parameter describing the migration rate, which is interpreted as the influence of drift and dispersal; m.ci, m.mle and maxLL were the fitting parameters of m; Rsqr, RMSE, AIC and BIC are used to assess the overall fit of the model to observed data; Richness is used to assess the number of species of each species pool; %AbovePred and %BelowPred represent the proportion of OTUs that occurs more or less frequently than that predicted by the neutral model, respectively.
