# Supplementary material for: Coupling Bacterial Community Assembly to Microbial Metabolism across Soil Profiles
Source: mSystems. 2020 Jun 9;5(3):e00298-20. doi: 10.1128/mSystems.00298-20 (PMC7289589; doi:10.1128/mSystems.00298-20)
Supplement: TABLE S3 [file mSystems.00298-20-st003.pdf]

Table S3 Topological properties of the bacterial co-occurrence networks across soil profiles.

	0−10 cm	10−20 cm	20−40 cm	40−60 cm	60−80 cm
Number of nodes	191	212	152	70	124
Number of edges	400	368	216	102	124
Percentage of negative correlations (PNC)	71.8	75.5	60.2	41.4	53.2
Average degree (*avgK*)	4.188	3.472	2.842	1.373	2.000
Average path length (APL)	2.648	2.644	2.481	1.236	1.740
Graph Density	0.022	0.016	0.019	0.014	0.016
Network diameter	7	7	6	3	4
Average clustering coefficient (*avgCC*)	0.025	0.022	0.016	0.004	0.009
Average Weighted degree	0.013	0.007	0.006	0.003	0.004
Modularity (M)	0.569	0.642	0.691	0.836	0.791
