# Supplementary material for: Coupling Bacterial Community Assembly to Microbial Metabolism across Soil Profiles
Source: mSystems. 2020 Jun 9;5(3):e00298-20. doi: 10.1128/mSystems.00298-20 (PMC7289589; doi:10.1128/mSystems.00298-20)
Supplement: TABLE S4 [file mSystems.00298-20-st004.pdf]

Table S4 The co-occurring incidence for bacterial intra-phyla was expected by random association in five soil layers. The observed co-occurring incidence (O) of the dominant phyla estimated by the relative percentage of the number of observed edges between them in respective network, while the random co-occurring incidence (R) was theoretically calculated by considering the phylum/class frequencies (F(Node1), F(Node2)) and random association[a].

	0–10 cm Nodes = 191, Edges = 400					
	F(N1)	F(N2)	Edges	O (%)	R (%)	O/R
Acidobacteria	37	37	24	6.00	3.67	**1.63**
Actinobacteria	46	46	26	6.50	5.70	**1.14**
Alphaproteobacteria	25	25	8	2.00	1.65	**1.21**
Betaproteobacteria	10	10	0	0.00	0.25	0.00
Chloroflexi	30	30	17	4.25	2.40	**1.77**
Deltaproteobacteria	11	11	0	0.00	0.30	0.00
Firmicutes	3	3	0	0.00	0.02	0.00
Gammaproteobacteria	7	7	1	0.25	0.12	**2.16**

	10–20 cm Nodes = 212, Edges = 368					
	F(N1)	F(N2)	Edges	O (%)	R (%)	O/R
Acidobacteria	41	41	19	5.16	3.67	**1.41**
Actinobacteria	23	23	3	0.82	1.13	0.72
Alphaproteobacteria	17	17	2	0.54	0.61	0.89
Betaproteobacteria	7	7	0	0.00	0.09	0.00
Chloroflexi	84	84	65	17.66	15.59	**1.13**
Deltaproteobacteria	8	8	0	0	0.13	0.00
Firmicutes	11	11	3	0.82	0.25	**3.32**
Gammaproteobacteria	4	4	0	0.00	0.03	0.00

	20–40 cm Nodes = 152, Edges = 216					
	F(N1)	F(N2)	Edges	O (%)	R (%)	O/R
Acidobacteria	24	24	3	3.24	2.41	0.58
Actinobacteria	19	19	3	1.39	1.49	0.93
Alphaproteobacteria	16	16	1	0.46	1.05	0.44
Betaproteobacteria	2	2	0	0.00	0.01	0.00
Chloroflexi	56	56	36	16.67	13.42	**1.24**
Deltaproteobacteria	6	6	0	0.00	0.13	0.00
Firmicutes	9	9	0	0.00	0.31	0.00
Gammaproteobacteria	2	2	0	0.00	0.01	0.00

	40–60 cm Nodes = 70, Edges = 102					
	F(N1)	F(N2)	Edges	O (%)	R (%)	O/R
Acidobacteria	20	20	2	2.86	3.76	0.75
Actinobacteria	15	15	1	1.43	2.08	0.69
Alphaproteobacteria	12	12	0	0.00	1.31	0.00
Betaproteobacteria	0	0	0	0.00	0.00	0.00
Chloroflexi	33	33	7	17.14	9.82	0.95
Deltaproteobacteria	6	6	0	0.00	0.30	0.00
Firmicutes	3	3	0	0.00	0.06	0.00
Gammaproteobacteria	4	4	0	0.00	0.12	0.00

	60–80 cm Nodes = 124, Edges = 124					
	F(N1)	F(N2)	Edges	O (%)	R (%)	O/R
Acidobacteria	25	25	2	1.61	3.93	0.41
Actinobacteria	25	25	1	0.81	3.93	0.21
Alphaproteobacteria	9	9	2	1.61	0.47	**3.42**
Betaproteobacteria	4	4	0	0.00	0.08	0.00
Chloroflexi	34	34	8	7.26	6.92	0.87
Deltaproteobacteria	7	7	1	0.81	0.28	**2.93**
Firmicutes	6	6	0	0.00	0.20	0.00
Gammaproteobacteria	3	3	0	0.00	0.04	0.00

a. The observed co-occurring incidences of intra-phyla higher than would be expected by chance (O/R > 1) was bold.
